# Taste for discovery: a conversation with neuroscientist Serge Charpak

**DOI:** 10.1117/1.NPh.11.1.010401

**Published:** 2024-01-10

**Authors:** Jérôme Lecoq

**Affiliations:** Allen Institute, Seattle, Washington, United States

## Abstract

Serge Charpak (Institut de la Vision) discusses his pioneering work in imaging of sensory processing and neurovascular coupling, in an interview with former trainee and fellow Neurophotonics Editorial Board Member Jérôme Lecoq (Allen Institute).

**Figure:**
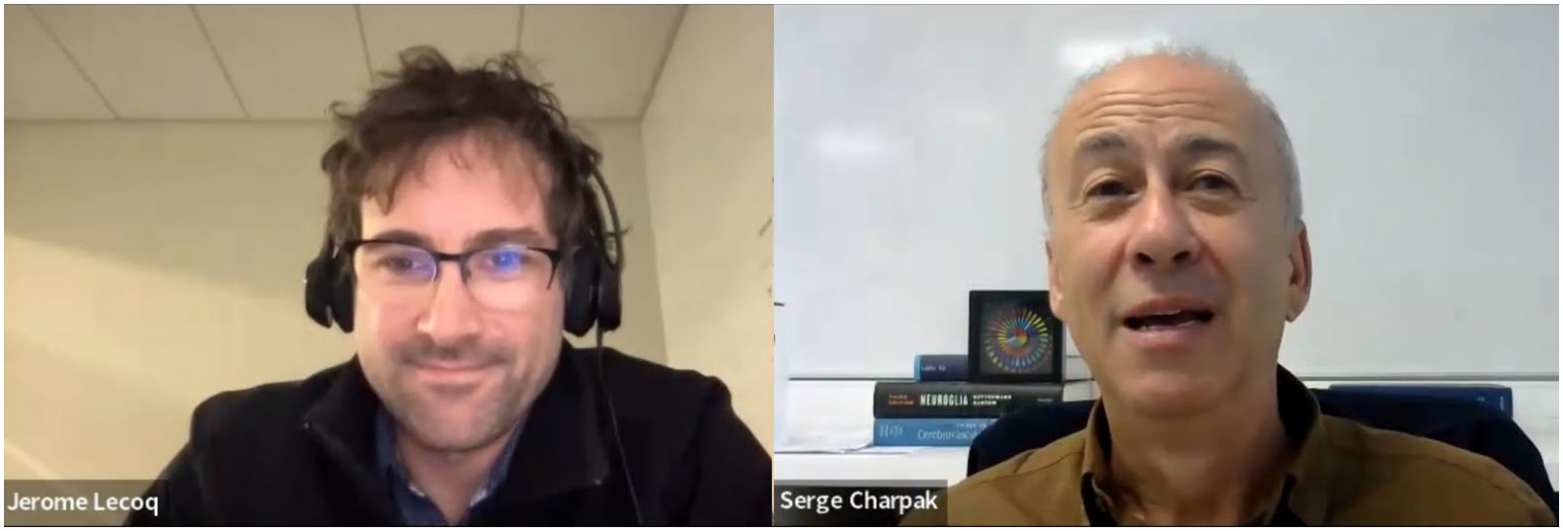
(Right) Serge Charpak (Institut de la Vision) discusses his pioneering work in imaging of sensory processing and neurovascular coupling, in an interview with former trainee and fellow *Neurophotonics* Editorial Board Member Jérôme Lecoq (Allen Institute). View a video recording of the interview at https://doi.org/10.1117/1.NPh.11.1.010401

